# Cannabinoids Regulate Bcl-2 and Cyclin D2 Expression in Pancreatic β Cells

**DOI:** 10.1371/journal.pone.0150981

**Published:** 2016-03-11

**Authors:** Jihye Kim, Kyung Jin Lee, Jung Seok Kim, Jun Gi Rho, Jung Jae Shin, Woo Keun Song, Eun Kyung Lee, Josephine M. Egan, Wook Kim

**Affiliations:** 1 Department of Molecular Science and Technology, Ajou University, Suwon, 16499, South Korea; 2 Department of Convergence Medicine, Asan Institute for Life Sciences, University of Ulsan College of Medicine, Asan Medical Center, Seoul, 05505, Korea; 3 Department of Life Science, Bio Imaging and Cell Dynamics Research Center, Gwangju Institute of Science and Technology, Gwangju, 61005, South Korea; 4 Department of Biochemistry, College of Medicine, The Catholic University of Korea, Seoul, 06591, South Korea; 5 Laboratory of Clinical Investigation, National Institute on Aging, National Institutes of Health, Baltimore, MD, 21224, United States of America; Sungkyunkwan University, REPUBLIC OF KOREA

## Abstract

Recent reports have shown that cannabinoid 1 receptors (CB1Rs) are expressed in pancreatic β cells, where they induce cell death and cell cycle arrest by directly inhibiting insulin receptor activation. Here, we report that CB1Rs regulate the expression of the anti-apoptotic protein Bcl-2 and cell cycle regulator cyclin D2 in pancreatic β cells. Treatment of MIN6 and βTC6 cells with a synthetic CB1R agonist, WIN55,212–2, led to a decrease in the expression of Bcl-2 and cyclin D2, in turn inducing cell cycle arrest in G0/G1 phase and caspase-3-dependent apoptosis. Additionally, genetic deletion and pharmacological blockade of CB1Rs after injury in mice led to increased levels of Bcl-2 and cyclin D2 in pancreatic β cells. These findings provide evidence for the involvement of Bcl-2 and cyclin D2 mediated by CB1Rs in the regulation of β-cell survival and growth, and will serve as a basis for developing new therapeutic interventions to enhance β-cell function and growth in diabetes.

## Introduction

The number of people diagnosed with diabetes worldwide has increased exponentially. However, it is currently not possible to directly treat the cause of diabetes. Type 1 diabetes (T1D) results from β-cell destruction by an autoimmune reaction that leads to insulin deficiency, and type 2 diabetes (T2D) is caused by insulin resistance and β-cell failure [1]. Therefore, insufficient insulin secretion due to β-cell loss is the common and major component in the pathogenesis of T1D and T2D, and decreased β-cell survival and growth are the primary mechanisms for β-cell loss [1]. The β-cell mass, which is governed by balancing β-cell death and proliferation, plays an essential role in maintaining optimal glucose homeostasis by determining the amount of insulin that is secreted into blood. Therefore, identifying the parameters that regulate β-cell death and proliferation and understanding their molecular mechanisms are especially important, and many molecules and signaling pathways have been identified. Among them, cyclin D2 and Bcl-2 are essential molecules in the regulation of β-cell growth and survival. Cyclin D2 is an essential regulator of β-cell expansion and stimulates cell cycle progression from G1 to S phase. Additionally, cyclin D2-deficient mice showed reduced β-cell growth and glucose intolerance [2–4]. The anti-apoptotic protein Bcl-2 is an essential molecule in the regulation of β-cell death. An imbalance between pro-apoptotic and anti-apoptotic Bcl-2 family proteins causes β-cell death via the mitochondrial pathway, and overexpression of Bcl-2 protects β cells from cytokine- and lipotoxic stress-induced cell death [5–7].

Because insulin is a key hormone that regulates not only energy homeostasis but also β-cell proliferation and death [8–10], many studies have focused on identifying factors that influence the insulin signaling pathway. Our recent studies have shown that the cannabinoid 1 receptor (CB1R), a G protein-coupled receptor that is activated by endogenous cannabinoids (ECs), is present in pancreatic β cells, in which its activation directly inhibits insulin receptor kinase activity by binding to the tyrosine kinase domain of the insulin receptor [11,12]. Activation of CB1Rs by ECs and synthetic cannabinoids induce β-cell death and cell cycle arrest by inhibiting insulin receptor signaling via IRS2-AKT-BAD and IRS2-AKT-p27, respectively [11,12]. Additionally, it has been reported that CB1Rs induce cell cycle arrest and death by inhibiting the PI3K-AKT cascade in various types of cancer cells [13–15]. Furthermore, treatment of cancer cells with the synthetic cannabinoid WIN55,212–2 led to the dose-dependent down-regulation of both cyclin D2 and Bcl-2 [15]. However, whether CB1Rs influence β-cell growth and survival by regulating the levels of cyclin D2 and Bcl-2 remains unclear. Here, we demonstrate that CB1R activation induces β-cell death and cell cycle arrest at G1 phase by decreasing Bcl-2 and cyclin D2 levels, respectively, both *in vitro* and *in vivo*.

## Materials and Methods

### Materials and Reagents

AM251(N-(piperidin-1-yl)-5-(4-iodophenyl)-1-(2,4-dichlorophenyl)-4-methyl-1H-pyrazole-3-carboxamide),WIN55,212-2([(3R)-2,3-dihydro-5-methyl-3-(4 morpholinylmethyl)pyrrolo[1,2,3-de]-1,4-benzoxazin-6-yl]-1-naphthalenyl-methanone) and streptozotocin (STZ) (2-deoxy-2-[[(methylnitrosoamino)carbonyl] amino]-D-glucose) were purchased from Cayman Chemical. Rimonabant (biaryl pyrazole N-(piperidinyl)-5-(4-chlorophenyl)-1-(2,4-dichlorophenyl)-4-methyl-1H-pyrazole-3-carboxamide) was synthesized at Ajou University. Antibodies against caspase-3, cyclin D2, and Bcl-2 were purchased from Cell Signaling Technology. An antibody against β-actin was purchased from Abcam. Antibody against Bax was purchased from Santa Cruz. Anti-mouse and anti-rabbit secondary horseradish peroxidase conjugate (HRP) was obtained from Bio-Rad Laboratories.

### Cell Culture and Treatment

The mouse pancreatic β cell lines MIN6 and βTC6 were cultured in Dulbecco’s modified Eagle’s medium supplemented with 10% heat-inactivated fetal bovine serum and 1% antibiotic penicillin and streptomycin and were maintained under standard cell culture conditions at 37°C and 5% CO2 in a humid environment. Cells were treated with WIN55,212–2 at 0, 1, 2.5, and 5 μM for 24 or 48 h in DMEM with 0.5% FBS with or without AM251.

### Cell Viability Assay

Cell viability was determined by MTS (3-(4,5-dimethylthiazol-2-yl)-5-(3-carboxymethoxyphenyl)-2-(4-sulphenyl)-2H- tetrazolium, inner salt) assay (Cell Titer 96 AQueous One Solution Cell Proliferation Assay; Promega). MIN6 and βTC6 cells were plated on 96-well plates and allowed to adhere to the plates overnight. Cells were treated with WIN-55,212–2 (0, 1, 2.5, 5 μM) for 24 h, followed by incubation with MTS dye for 2 h. Absorbance was determined at 490 nm using iMark (Bio-Rad).

### Western Blot Analysis

Whole cell lysates were prepared using modified radioimmune precipitation assay buffer (10 mM Tris-HCl, pH 7.4, 150 mM NaCl, 1% NonidetP-40, 1 mM EDTA, and 0.1% SDS), separated by electrophoresis in SDS-containing polyacrylamide gels, and transferred onto PVDF membranes (Millipore). Incubation with primary antibodies against caspase-3, cyclin D2, Bcl-2, Bax, and β-actin was followed by incubations with the appropriate secondary antibodies conjugated with HRP.

### Cell Cycle Analysis by Flow Cytometry

Cells were plated at a density of 1x10^6^ cells on 6-well culture dishes. After an overnight incubation, cells were treated with WIN-55,212–2 (1.0, 2.5, 5.0 μM) for 48 h, washed twice with cold phosphate-buffer saline (PBS), detached with 0.25% trypsin-EDTA, and pelleted. The pellet was suspended in cold PBS, fixed in a final concentration of 70% ethanol for 1 h at 4°C, washed with cold PBS, and incubated with 100 μg/ml RNase A for 15 min at room temperature. Nuclei were stained with 50 μg/ml propidium iodide (PI; Sigma-Aldrich) for 30 min at 4°C in the dark. Samples were analyzed by flow cytometry using a fluorescence activated cell sorter (FACS). Results were analyzed using Mod-Fit LT software (Verity Software House, Topsham, ME) to determine cell cycle distribution.

### Animal Experiments

All animal experiments were carried out in compliance with the protocol specifically approved for this study by the Ajou University Animal Care and Use Committee. *CB1R−/−* mice and their wild-type littermates were developed and backcrossed into a C57Bl/6J background, as previously described [16]. For regeneration experiments, low-dose (50 mg/kg) STZ was administered by daily i.p. injection into 2-month-old CD1 (Fig A in S1 File) or *CB1R−/−* and *CB1R+/+* mice (n = 5 per group) for 5 days (Fig B in S1 File). DMSO or AM251 (10 mg/kg) was then administered into CD1 mice by daily i.p injection without STZ. Three weeks after STZ withdrawal, mice were euthanized with a lethal dose of isoflurane and pancreata were collected for the metabolic and morphological analyses. DMSO or rimonabant (5 mg/kg) was administrated by daily intraperitoneal (i.p.) injection into 6-week-old *db/db* mice (n = 5 per group) for 4 weeks (Fig C in S1 File). Pancreata were rapidly dissected, fixed, and sectioned at a thickness of 7 μm. After antigen unmasking, the slides were blocked with 5% bovine serum albumin (BSA)/PBS and incubated at 4°C with a specific primary antibody, followed by secondary antibodies along with DAPI, in some cases, for nuclear staining. Slides were viewed with a Leica DMRBE microscope equipped with a 400X objective lens. Signal intensity was assessed using ImageJ software (http://rsb.info.nih.gov/ij/).

### Statistical Analysis

Quantitative data are presented as the mean ± SEM. Differences between mean values were compared statistically by Student’s *t*-test. Comparisons were performed using GraphPad Prism (GraphPad Software) and Microsoft Excel 2010. A *P* value < 0.05 was considered statistically significant.

## Results and Discussion

### Activation of CB1R with WIN55,212–2 leads to decreased β cell survival

**Fig 1:**
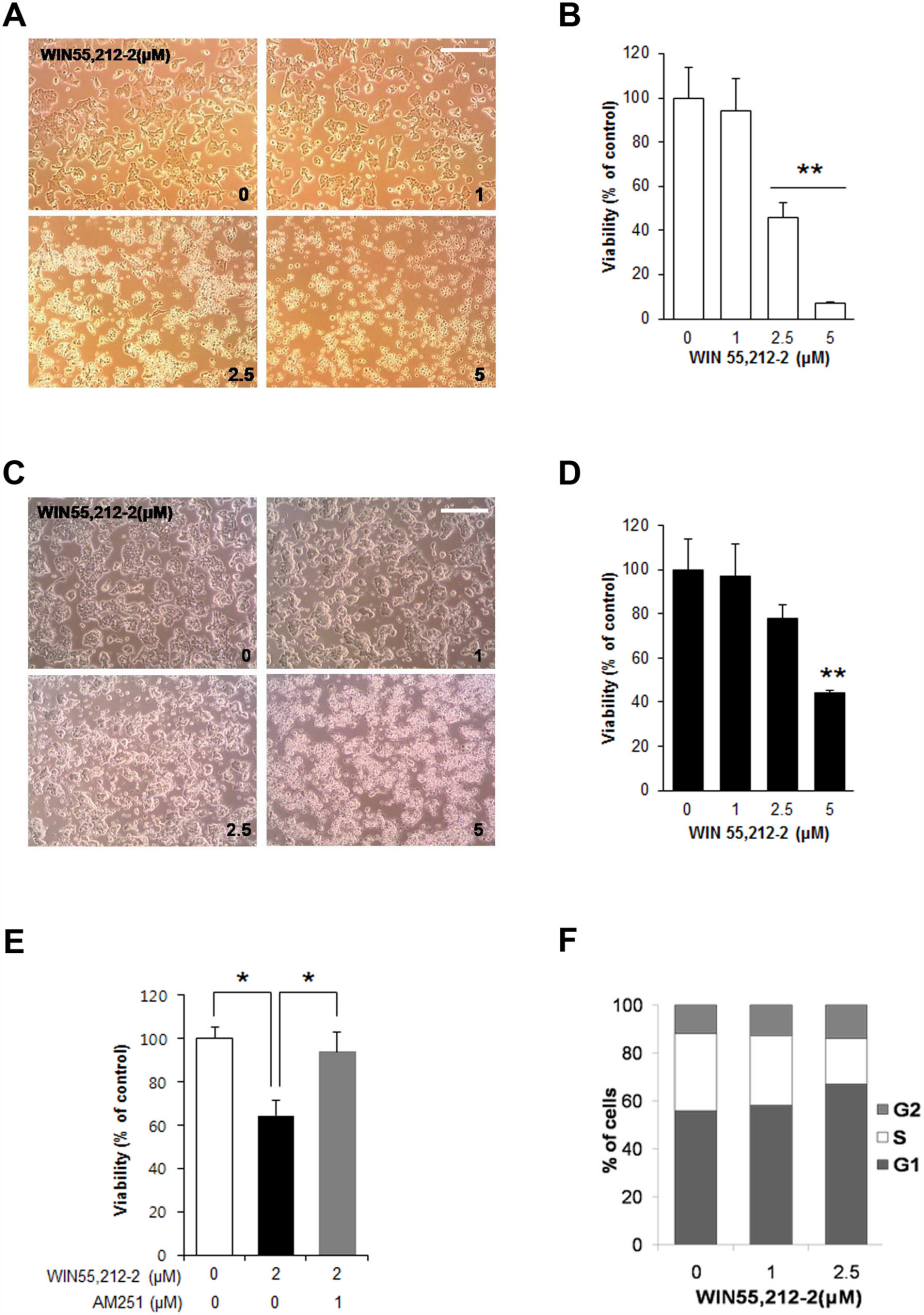
The synthetic CB1R agonist WIN55,212–2 leads to decreased cell viability and increased G1 arrest in pancreatic β-cell lines. (A) Representative images of MIN6 cells exposed to WIN55,212–2. Scale bar, 50 μm. (B) The relative cell viability of MIN6 cells was determined by MTS assay 24 h after treatment with WIN55,212–2. Scale bar, 50 μm. (C) Representative images of βTC6 cells exposed to WIN55,212–2. (D) The relative cell viability of βTC6 cells was determined by MTS assay 24 h after treatment with WIN55,212–2. (E) The relative cell viability of MIN6 cells exposed to WIN55,212–2 with or without a CB1R inverse agonist AM251. MIN6 cells were exposed to WIN55,212–2 with or without AM251 for 24 h, and relative cell viability was determined by MTS assay. (F) WIN55,212–2 treatment in βTC6 cells increases the proportion of cells in G0/G1 phase. βTC6 cells exposed to WIN55,212–2 for 24 h were analyzed using a fluorescence activated cell sorter (FACS). Data are shown as the mean ± SEM from three independent experiments. *P< 0.05; **P< 0.01.

We first investigated the effects of the synthetic CB1R agonist WIN55,212–2 on pancreatic β-cell viability. Treatment of mouse insulinoma MIN6 cells with WIN55,212–2 caused morphological changes characteristic of apoptosis, including cell rounding, in a dose-dependent manner (Fig 1A). Additionally, the viability of MIN6 cells dose-dependently decreased with WIN55,212–2 treatment (Fig 1B). Similar effects of WIN55,212–2 on morphological changes (Fig 1C) and viability (Fig 1D) were observed in another mouse insulinoma cell line (βTC6). The effects of WIN55,212–2 on viability were prevented by AM251, a selective CB1R antagonist (Fig 1E), suggesting that these effects are mediated by CB1Rs. We next examined whether WIN55,212–2 regulates cell cycle progression in pancreatic β-cell lines. To analyze cell cycle distribution, DNA content analysis was performed with PI staining and subsequent FACS analysis after treatment with WIN55,212–2. As shown in Fig 1F, treatment of βTC6 cells with WIN55,212–2 led to an increase in the proportion of cells in G1 phase and a decrease in the proportion of cells in S phase. These results indicate that activation of CB1R by WIN55,212–2 induces cell cycle arrest at G1 phase in pancreatic β cells.

### WIN55,212–2 decreases Bcl-2 and cyclin D2 expression in pancreatic β cells

**Fig 2:**
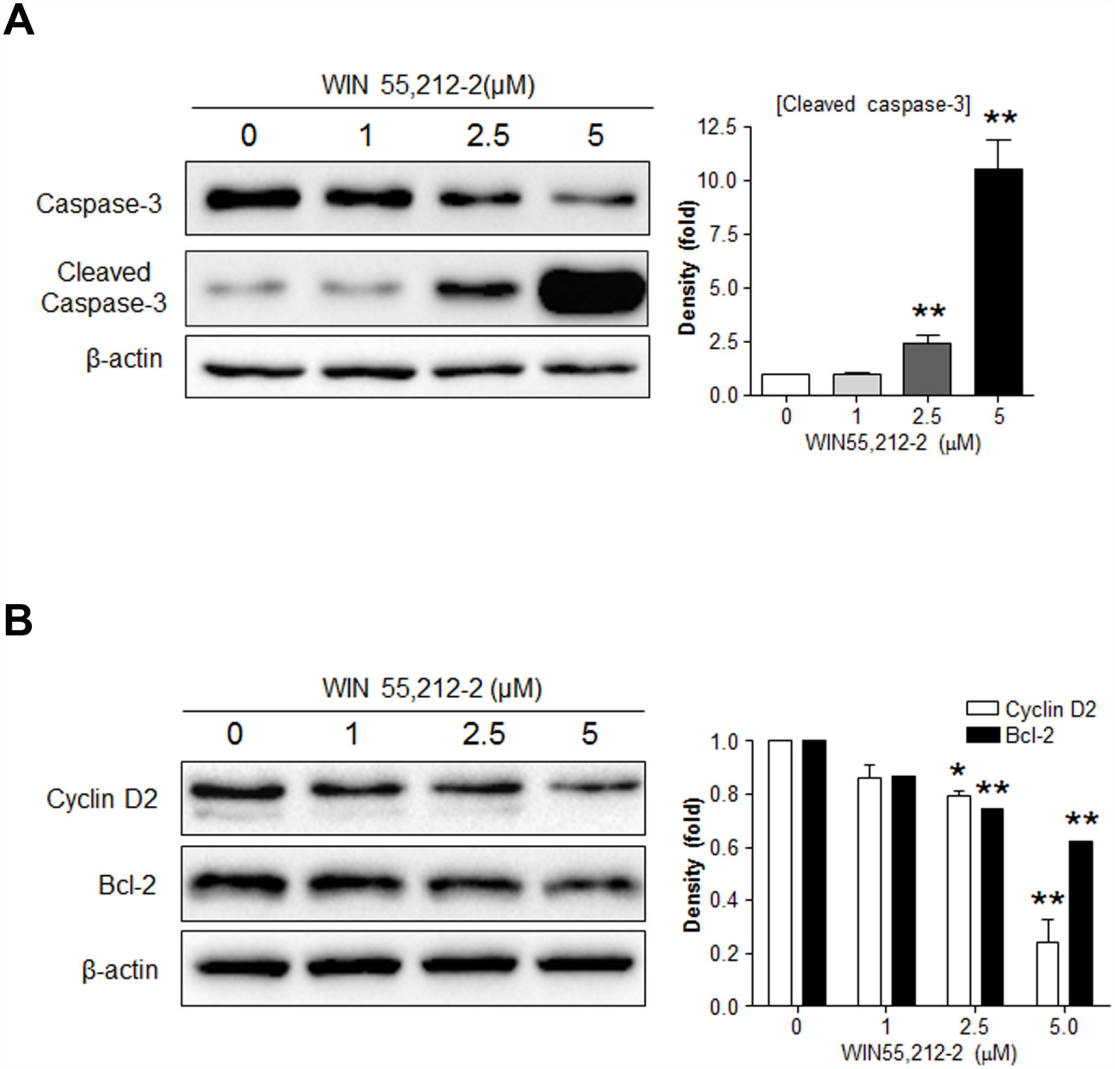
Effects of WIN55,212–2 treatment on the expression of Bcl-2 and cyclin D2 in βTC6 cells. βTC6 cells exposed to WIN55,212–2 for 24 h were subjected to western blot analysis for cleaved caspase-3 (A) or cyclin D2 and Bcl-2 (B). Relative densities for the indicated proteins are shown on the right. Data are shown as the mean ± SEM from three independent experiments. *P< 0.05; **P< 0.01.

Because cyclin D2 and Bcl-2 are pivotal molecules in the regulation of β-cell growth and survival [2–7], we next investigated the potential role of these molecules as a/the mediator of WIN55,212-2-controlled β-cell growth and survival. Consistent with our previous results (Fig 1), WIN55,212–2 induced caspase-3 activation in βTC6 cells in a dose-dependent manner (Fig 2A). WIN55,212–2 caused a dose-dependent decrease in the levels of both cyclin D2 and Bcl-2 in βTC6 cells (Fig 2B). Similar effects of WIN55,212–2 on caspase-3 activation and cyclin D2 and Bcl-2 levels were also observed in MIN6 cells (data not shown). These results suggest that WIN55,212-2-induced cell cycle arrest and cell death might be mediated by decreasing cyclin D2 and Bcl-2 levels, respectively.

### CB1R blockade in diabetic mouse models increases Bcl-2 and cyclin D2 levels in pancreatic β cells

**Fig 3:**
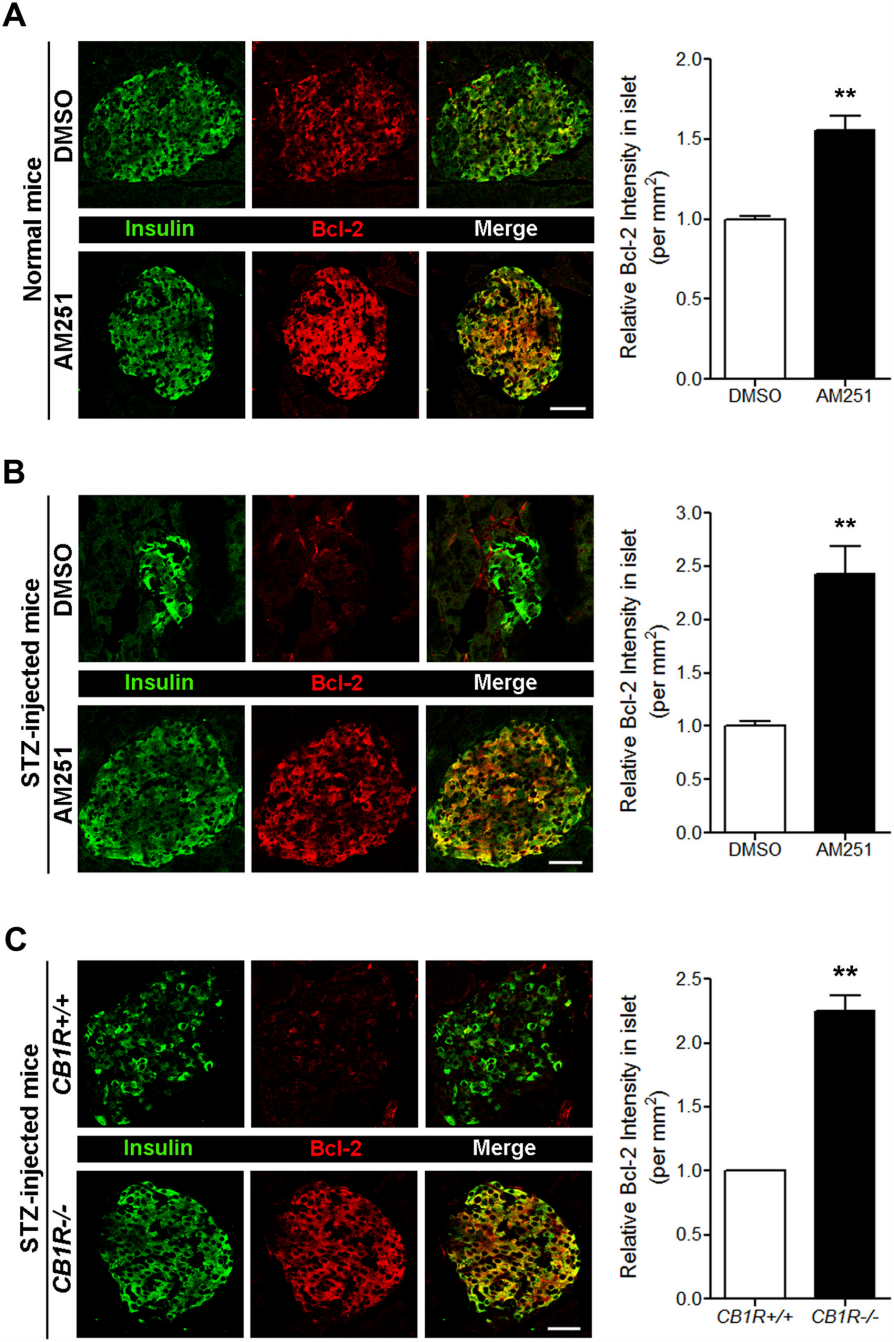
Blockade of CB1Rs in normal and STZ-treated mice increases Bcl-2 levels in β cells. (A) Representative images for insulin and Bcl-2 in β cells of DMSO- and AM251-injected normal mice. The relative signal intensity for Bcl-2 in β cells is shown on the right. (B) Representative images for insulin and Bcl-2 in β cells of DMSO- and AM251-injected mice after STZ treatment. The relative signal intensity for Bcl-2 in β cells is shown on the right. (C) Representative images for insulin and Bcl-2 in β cells of *CB1R+/+* and *CB1R-/-* mice 4 weeks after STZ treatment. The relative signal intensity for Bcl-2 in β cells is shown on the right. Data are shown as the mean ± SEM from n = 3–5 animals per group. *P< 0.05; **P< 0.01. Scale bar, 50 μm.

**Fig 4:**
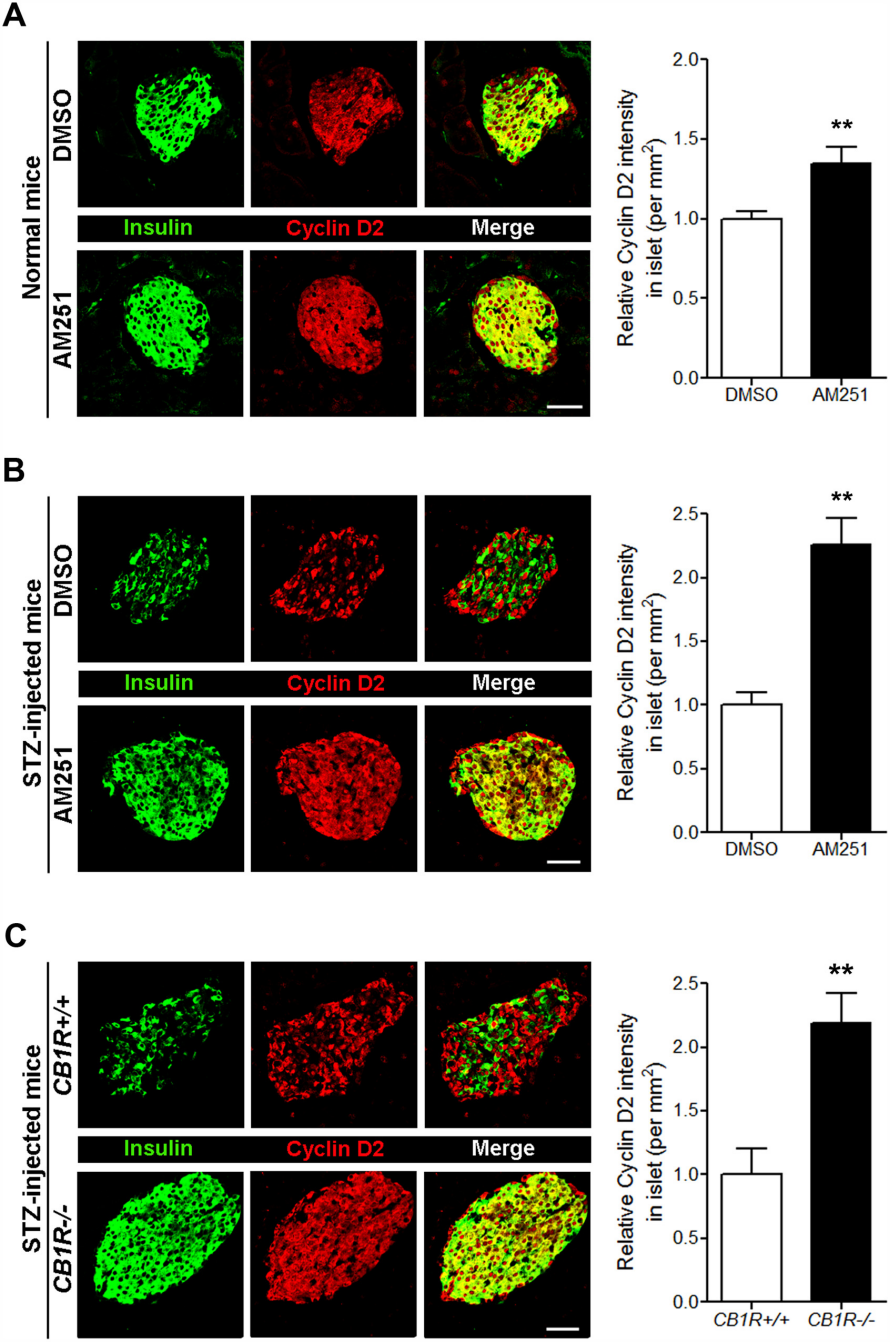
Blockade of CB1Rs in normal and STZ-treated mice increases cyclin D2 levels in β cells. (A) Representative images for insulin and cyclin D2 in β cells of DMSO- and AM251-injected normal mice. The relative signal intensity for cyclin D2 in β cells is shown on the right. (B) Representative images for insulin and cyclin D2 in β cells of DMSO- and AM251-injected mice after STZ treatment. The relative signal intensity for cyclin D2 in β cells is shown on the right. (C) Representative images for insulin and cyclin D2 in β cells of *CB1R+/+* and *CB1R-/-* mice 4 weeks after STZ treatment. The relative signal intensity for cyclin D2 in β cells is shown on the right. Data are shown as the mean ± SEM from n = 3–5 animals per group. *P< 0.05; **P< 0.01. Scale bar, 50 μm.

Our previous report demonstrated that the pharmacological and genetic blockade of CB1Rs has beneficial effects on β-cell growth and survival in mouse models of T1D [12]. Multiple injections of low-dose STZ cause insulin-dependent diabetes and produce a progressive increase in blood glucose levels due to selective β-cell destruction, and over time, the remaining β cells attempt to survive and proliferate [17,18]. Compared with control mice, genetic deletion and pharmacological blockade of CB1Rs in STZ-injected mice lead to decreased blood glucose levels and increased β-cell growth and survival resulting from decreased levels of cyclin-dependent kinase inhibitor p27 and active caspase-3 caused by enhanced insulin signaling via the IRS2-AKT pathway [12]. Similar effects were also observed in AM251-injected normal and *db/db* mice [11]. Thus, using pancreata from those mice, we further examined whether the increased β-cell survival and growth seen following CB1R blockade in these mice are associated with any changes in the expression levels of Bcl-2 and cyclin D2. As shown in Fig 3A, daily injection of AM251 for 3 weeks in normal mice led to significantly increased levels of Bcl-2 in pancreatic β cells compared with DMSO-treated mice. Additionally, CB1R blockade by AM251 in STZ-injected mice increased the amount of Bcl-2 in pancreatic β cells compared with DMSO-treated mice (Fig 3B), and STZ-injected CB1R-null (*CB1R-/-*) mice also showed enhanced levels of Bcl-2 in pancreatic β cells compared with STZ-injected *CB1R+/+* mice (Fig 3C). Similar to Bcl-2, the levels of cyclin D2 in pancreatic β cells of normal mice were also significantly increased by AM251 (Fig 4A). Furthermore, CB1R blockade by AM251 in STZ-injected mice also significantly increased cyclin D2 levels in pancreatic β cells (Fig 4B), and a similar pattern was evident in pancreatic sections from STZ-injected *CB1R-/-* mice (Fig 4C).

**Fig 5:**
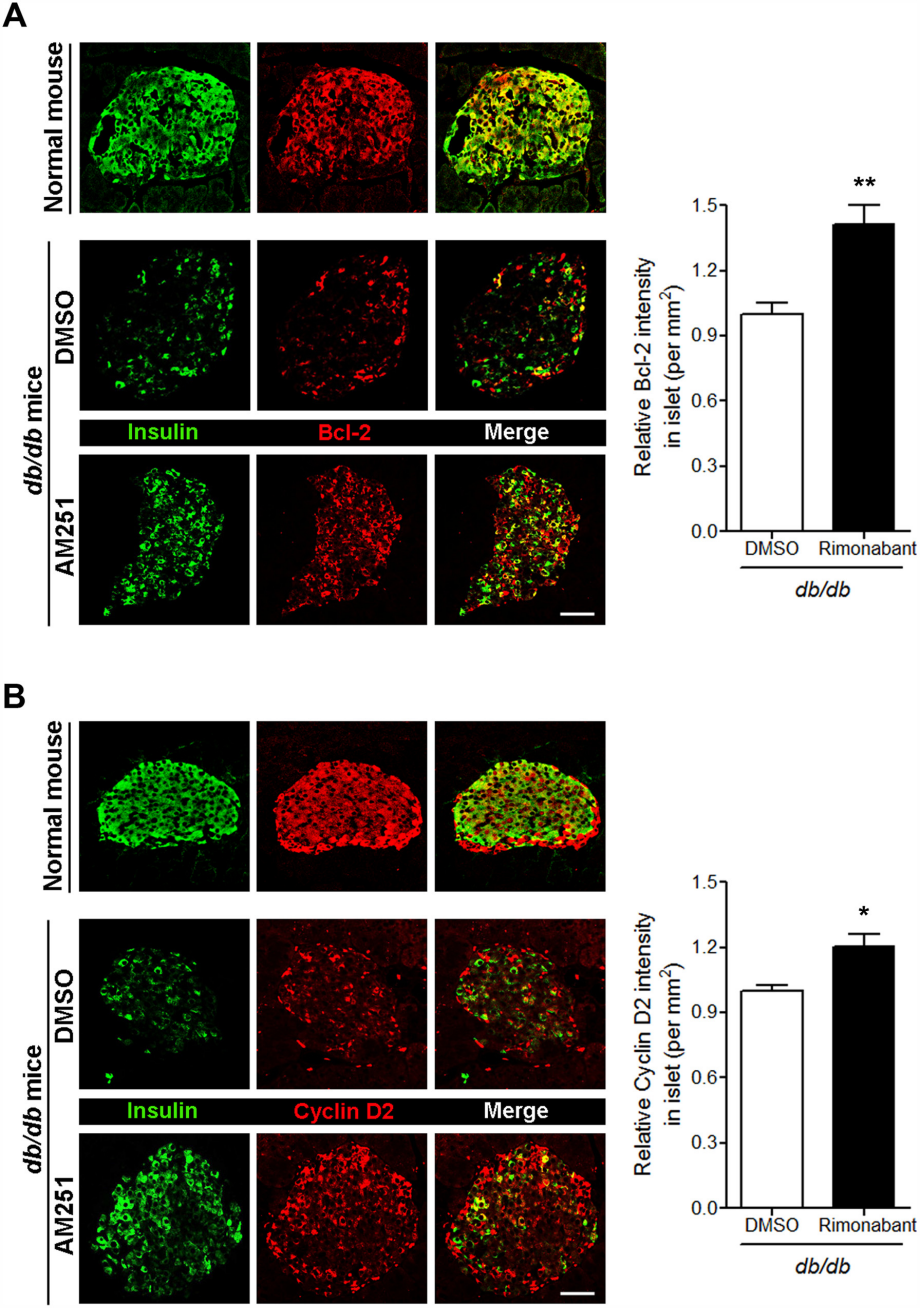
Blockade of CB1Rs in *db/db* mice increases Bcl-2 and cyclin D2 levels in β cells. (A) Representative images for insulin and Bcl-2 in β cells of normal and DMSO- or rimonabant-injected *db/db* mice. The relative signal intensity for Bcl-2 in β cells is shown on the right. (B) Representative images for insulin and cyclin D2 in β cells of normal and DMSO- or rimonabant-injected *db/db* mice. The relative signal intensity for cyclin D2 in β cells is shown on the right. Data are shown as the mean ± SEM from n = 5 animals per group. *P< 0.05; **P< 0.01. Scale bar, 50 μm.

The pharmacological blockade of CB1Rs in the T2D mouse model resulted in decreased blood glucose levels as well as increased intra-islet insulin content and β-cell mass due to enhanced β-cell proliferation [11]. Thus, we then investigated the expression levels of Bcl-2 and cyclin D2 in pancreatic β cells of *db/db* mice. The Bcl-2 level was reduced in pancreatic β cells of *db/db* mice compared with normal mice (Fig 5A). Daily injection of another selective CB1R antagonist (rimonabant) for 4 weeks in *db/db* mice led to significantly increased levels of Bcl-2 in pancreatic β cells compared with DMSO-treated mice (Fig 5A). A similar pattern for cyclin D2 was also observed in pancreatic β cells of rimonabant-injected *db/db* mice (Fig 5B). These results suggest that the effects of CB1R blockade on β-cell growth and survival are mediated, at least in part, by enhanced Bcl-2 and cyclin D2 levels.

CB1Rs play critical roles in regulation of β-cell mass and function by influencing insulin receptor signaling pathway [11,12,19]. Recent reports have shown that activation of CB1Rs by ECs and synthetic cannabinoids inhibited insulin receptor signaling, resulting in reduced β-cell mass [11,12]. The serine/threonine kinase AKT is the primary mediator of insulin receptor signaling and many downstream targets of AKT have been identified that may underlie the ability of this cascade to regulate β-mass. It has been well known that AKT directly phosphorylate several crucial downstream molecules that mediate cell survival and proliferation signals, such as pro-apoptotic protein BAD, forkhead transcription factor FoxO1, and cyclin-dependent kinase inhibitor p27, leading to the suppression of the cell death and growth arrest signals [20–24]. Consistently, our previous studies have shown that activation of CB1Rs induces β-cell death and cell cycle arrest by inhibiting AKT-mediated phosphorylation of BAD and p27, respectively [11,12]. Both cyclin D2 and Bcl-2 are also crucial downstream targets of AKT that play essential roles in regulation of β-cell mass [3–7,25,26]. Therefore, our data suggest that CB1Rs could regulate the expression of cyclin D2 and Bcl-2 by influencing insulin receptor signaling via IRS2-AKT cascade.

## Conclusions

In the present report, we provide evidence for a molecular mechanism by which CB1Rs regulate β-cell growth and survival through influencing cyclin D2 and Bcl-2. We found that activation of CB1R leads to a decrease in the expression of cyclin D2 and Bcl-2, in turn inducing cell cycle arrest in G0/G1 phase and caspase-3-dependent apoptosis both *in vitro* and *in vivo*. Taken together, these findings suggest involvement of Bcl-2 and cyclin D2 in the regulation of β-cell survival and growth by CB1Rs. Further studies examining the direct relationship between EC-induced β-cell death and Bcl-2 as well as EC-induced β-cell growth arrest and cyclin D2 are warranted, which would contribute to identify the exact mechanism by which CB1Rs regulate β-cell growth and survival.

## Supporting Information

S1 File Experimental timeline of the study. (TIF)
