# Supplementary figures and images for: Cannabinoids Regulate Bcl-2 and Cyclin D2 Expression in Pancreatic β Cells
Source: PLoS One. 2016 Mar 11;11(3):e0150981. doi: 10.1371/journal.pone.0150981 (PMC4788443; doi:10.1371/journal.pone.0150981)

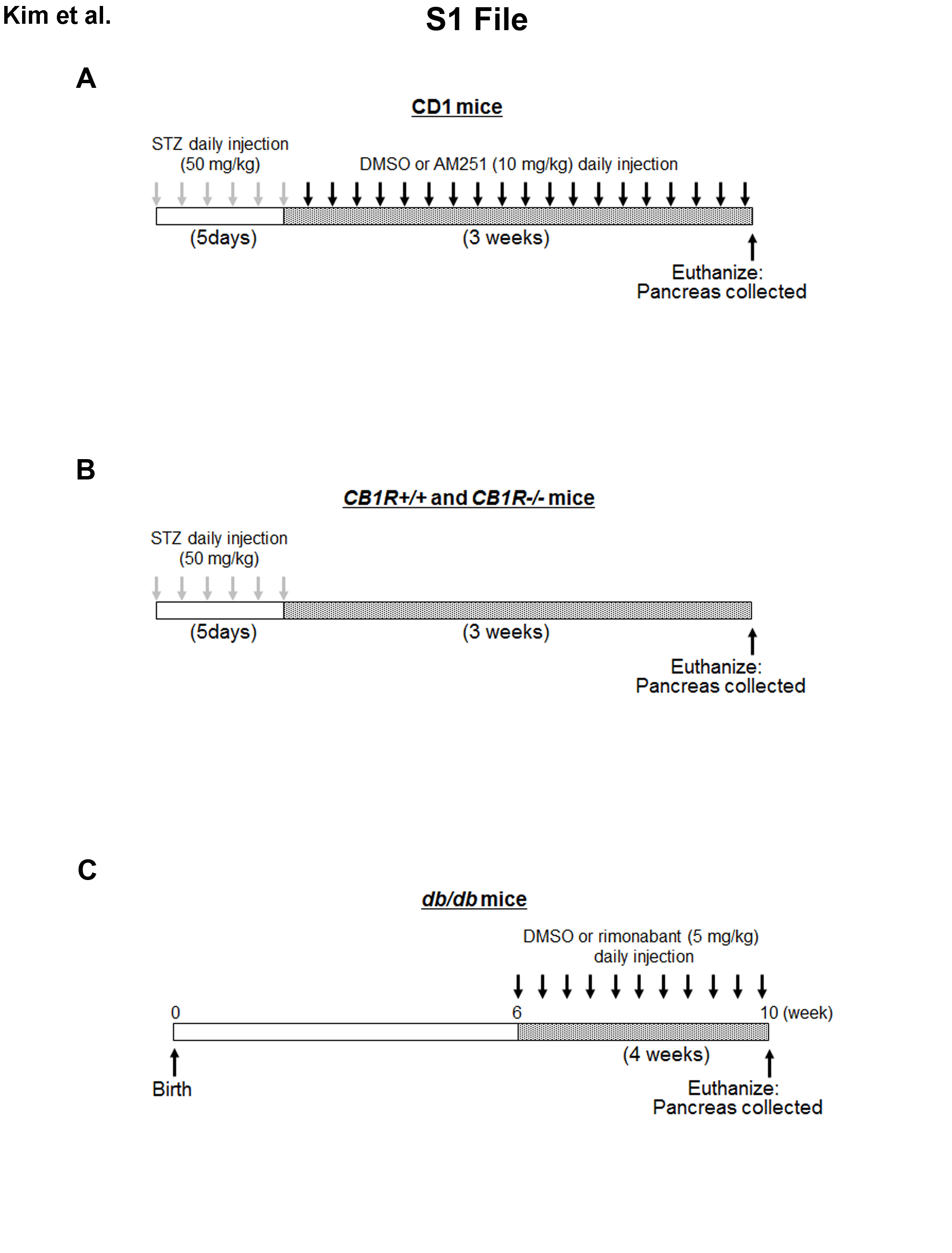

Supplement: S1 File — (TIF) [file pone.0150981.s001.tif]
